# A copper(II) paddle-wheel structure of tranexamic acid: di­chloro-tetra­kis­[μ-4-(ammonio­meth­yl)cyclo­hexane-1-carboxyl­ato-*O*,*O*′]dicopper(II) dichloride hexa­hydrate

**DOI:** 10.1107/S2056989017012543

**Published:** 2017-09-08

**Authors:** Muhammad Altaf, Helen Stoeckli-Evans

**Affiliations:** a Center of Excellence in Nanotechnology, King Fahd University of Petroleum, & Minerals, 31261 Dahran, Saudi Arabia; b Institute of Physics, University of Neuchâtel, rue Emile-Argand 11, CH-2000 Neuchâtel, Switzerland

**Keywords:** crystal structure, tranexamic acid, anti­fibrinolytic amino acid, fibrinolytic inhibitor, copper(II), paddle-wheel, hydrogen bonding

## Abstract

Tranexamic acid [systematic name: *trans*-4-(amino­meth­yl)cyclo­hexane-1-carb­oxy­lic acid], is an anti­fibrinolytic amino acid that exists as a zwitterion [*trans*-4-(ammonio­meth­yl)cyclo­hexane-1-carboxyl­ate] in the solid state. Its reaction with copper chloride leads to the formation of a compound with a copper(II) paddle-wheel structure that crystallizes as a hexa­hydrate, [Cu_2_Cl_2_(C_8_H_15_NO_2_)_4_]^2+^·2Cl^−^·6H_2_O. The asymmetric unit is composed of a copper(II) cation, two zwitterionic tranexamic acid units, a coordinating Cl^−^ anion and a free Cl^−^ anion, together with three water mol­ecules of crystallization. The whole structure is generated by inversion symmetry, with the Cu⋯Cu axle of the paddle-wheel dication being located about a center of symmetry. The cyclo­hexane rings of the zwitterionic tranexamic acid units have chair conformations. The carboxyl­ate groups that bridge the two copper(II) cations are inclined to one another by 88.4 (8)°. The copper(II) cation is ligated by four carboxyl­ate O atoms in the equatorial plane and by a Cl^−^ ion in the axial position. Hence, it has a fivefold O_4_Cl coordination sphere with a perfect square-pyramidal geometry and a *τ*_5_ index of zero. In the crystal, the paddle-wheel dications are linked by a series of N—H⋯Cl hydrogen bonds, involving the coordinating and free Cl^−^ ions, forming a three-dimensional network. This network is strengthened by a series of N—H⋯O_water_, O_water_—H⋯Cl and O_water_—H⋯O hydrogen bonds.

## Abstract 2

Tranexamic acid is an anti­fibrinolytic amino acid that exists as a zwitterion [*trans*-4-(ammonio­meth­yl)cyclo­hexane-1-carboxyl­ate] in the solid state. Its reaction with copper chloride lead to the formation of a copper(II) paddle-wheel structure.

## Chemical context   

**Figure 1:**
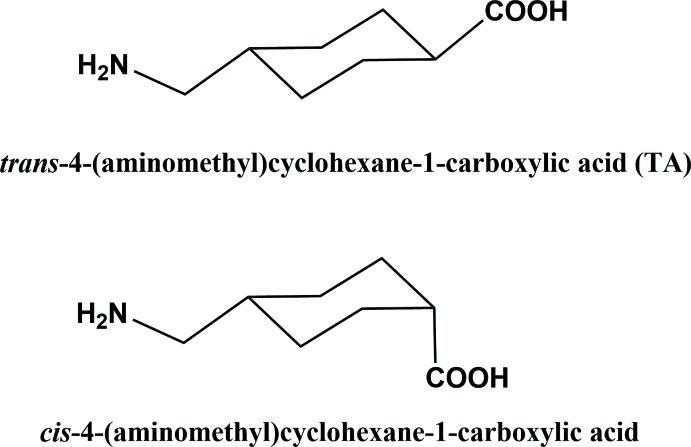
The *trans*- and *cis*-isomers of 4-(amino­meth­yl)cyclo­hexane-1-carb­oxy­lic acid.

Tranexamic acid (TA) is a derivative of the amino acid lysine. It has important anti­fibrinolytic activity and is used extensively in both trauma and normal surgery to prevent excessive blood loss (Napolitano *et al.*, 2013 ▸; Melvin *et al.*, 2015 ▸). It was first synthesized in the early 1960s by the Japanese husband and wife team Shosuke and Utako Okamato (1962 ▸). They showed amino-methyl-cyclo­hexane-carb­oxy­lic acid (AMCHA) to be a new inhibitor of fibrinolysis. Almost simultaneously with a Swedish group (Melander *et al.*, 1965 ▸), they were able to show that the anti­fibrinolytic active isomer (Okamoto *et al.*, 1964 ▸) has a *trans*-conformation (TA; Fig. 1 ▸) with the amino­methyl and carb­oxy­lic acid substituents on the cyclo­hexane ring occupying equatorial positions (Fig. 1 ▸). The *cis*-isomer (Fig. 1 ▸), in which the carb­oxy­lic acid moiety is axial, is almost inactive. The latter was shown to exist as the free acid in the solid state (Yamazaki *et al.*, 1981 ▸), in contrast to the *trans*-isomer, which exists as a zwitterion in the solid state (Groth, 1968 ▸; Shahzadi *et al.*, 2007 ▸). Recently, Tengborn *et al.* (2015 ▸) published an excellent review article, entitled ‘Tranexamic acid – an old drug still going strong and making a revival’, in which they recount the history of the development of TA and its mechanism of action, pharmokinetics and other details, including clinical uses. Herein, we report on the first crystal structure of a metal complex of tranexamic acid. The reaction of TA with copper(II) chloride leads to the formation of the title compound with a copper(II) paddle-wheel structure, that crystallizes as a hexa­hydrate. The reaction of TA with copper(II) bromide leads to the formation of an isotypical compound; however, the crystals were twinned and the subsequent X-ray analysis was of insufficient quality to be submitted or deposited.
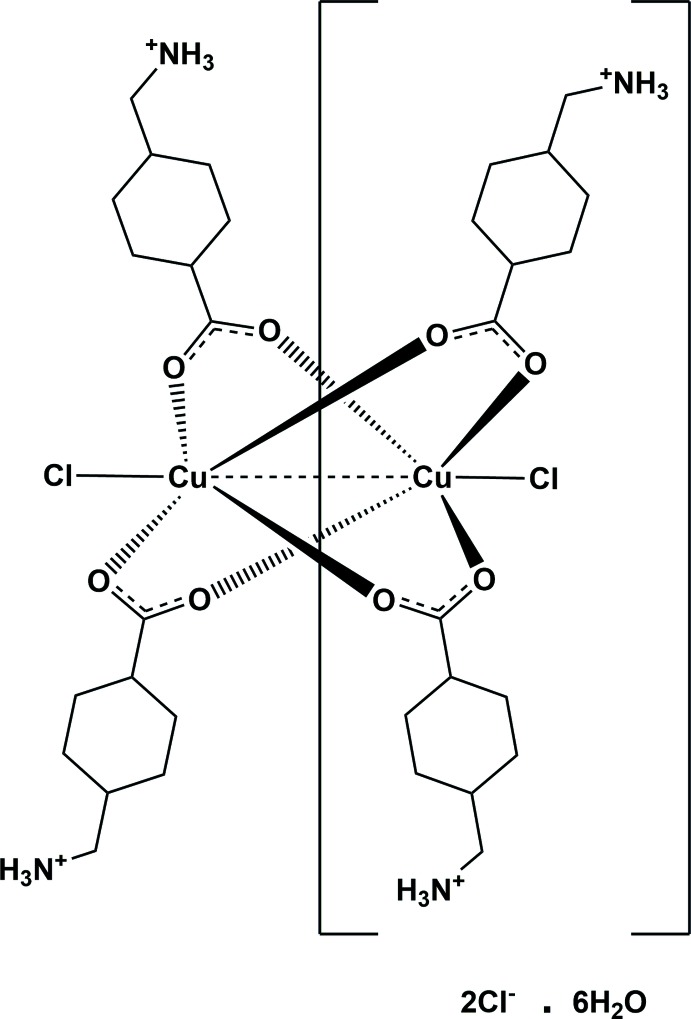


## Structural commentary   

**Figure 2:**
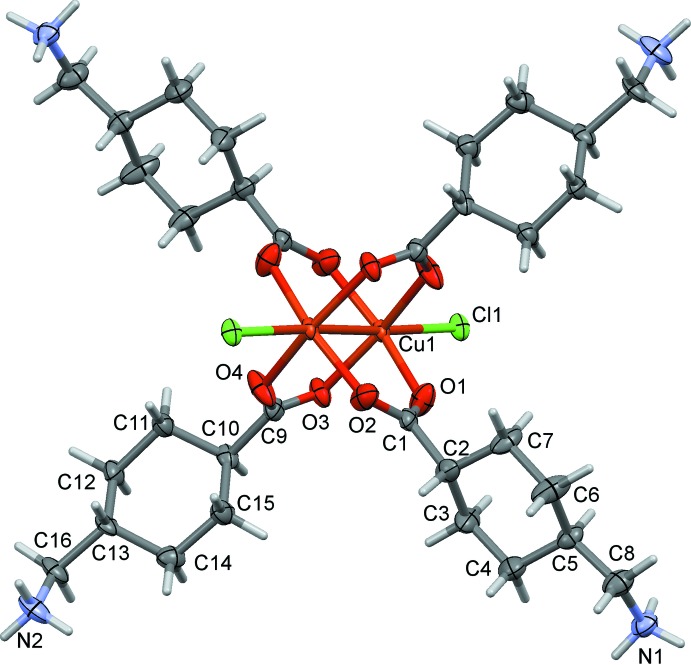
A view of the mol­ecular structure of the title dication, with atom labelling. Displacement ellipsoids are drawn at the 50% probability level. Unlabelled atoms are related to the labelled atoms by inversion symmetry (symmetry operation: −*x* + 1, −*y* + 1, −*z* + 1).

The mol­ecular structure of the dication of the title compound is illustrated in Fig. 2 ▸. The asymmetric unit is composed of a copper(II) cation coordinated by the carboxyl­ate O atoms (O1–O4) of two zwitterionic tranexamic acid units and a Cl^−^ anion, Cl1, together with a free Cl^−^ anion, Cl2, and three water mol­ecules of crystallization. The whole structure is generated by inversion symmetry, with the Cu1⋯Cu1^i^ axle [2.6649 (11) Å; symmetry code (i): −*x* + 1, −*y* + 1, −*z* + 1] of the paddle-wheel being located about a center of symmetry. Selected bond lengths and angles in the paddle-wheel dication are given in Table 1 ▸. Atom Cu1 is coordinated by four carboxyl­ate O atoms (O1–O4) in the equatorial plane and a Cl^−^ ion, Cl1, in the axial position. The Cu—O distances vary from 1.950 (4) to 1.991 (3) Å, with a longer Cu1—Cl1 axial distance of 2.499 (1) Å (Table 1 ▸). The copper(II) cation, Cu1 (Cu1^i^), has a perfect square-pyramidal coordination sphere with a *τ*_5_ index of 0.0 (*τ*_5_ = 0 for an ideal square-pyramidal coordination sphere, and = 1 for an ideal trigonal–pyramidal coordination sphere; Addison *et al.*, 1984 ▸).

The conformations of the two zwitterionic tranexamic acid units differ slightly. The cyclo­hexane rings (C2–C7 and C10–C15) have chair conformations; puckering parameters for ring C2–C7 are *Q* = 0.569 (7) Å, θ = 176.3 (6)°, φ = 358 (13)°, and for ring C10–C15 are *Q* = 0.568 (6) Å, θ = 6.0 (6)°, φ = 137 (6)°. The carboxyl­ate groups (C1/O1/O2 and C9/O3/O4) are inclined to the mean planes of the four planar atoms of the respective cyclo­hexane rings (C3/C4/C6/C7 and C11/C12/C14/C15) by 67.5 (6) and 85.8 (7)°, while they are inclined to one another by 88.4 (8)°. The ammonio­methyl units, C5/C8/N1 and C13/C16/N2, are inclined to the mean planes of the four planar atoms of the respective cyclo­hexane rings (C3/C4/C6/C7 and C11/C12/C14/C15) by 34.9 (6) and 47.5 (6)°.

## Supra­molecular features   

**Figure 3:**
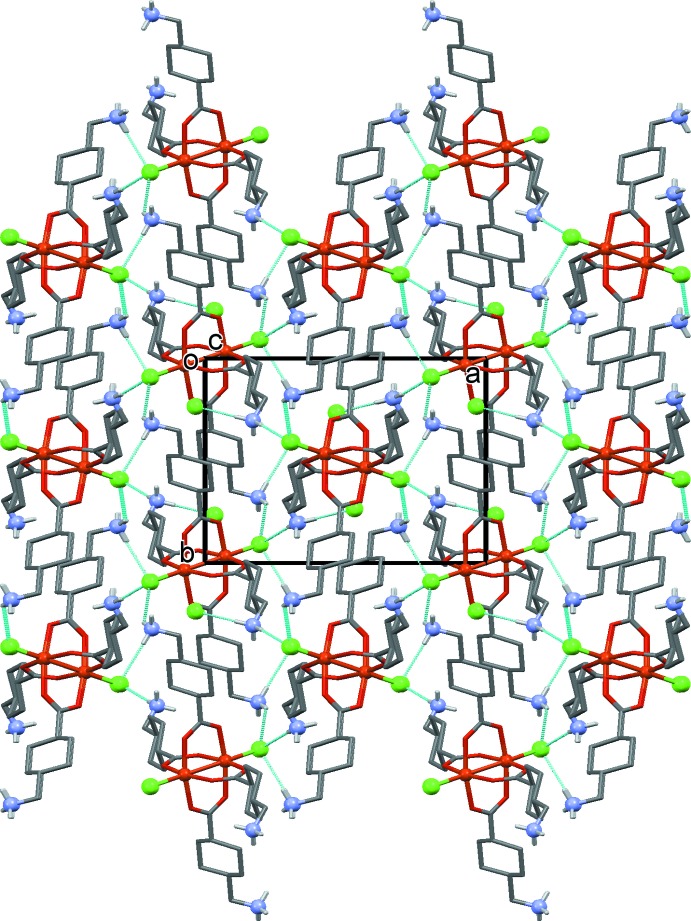
A view along the *c* axis of the crystal structure of the title compound with the water solvent mol­ecules omitted. The N—H⋯Cl hydrogen bonds are shown as dashed lines (see Table 2 ▸), and the C-bound H atoms have been omitted for clarity.

**Figure 4:**
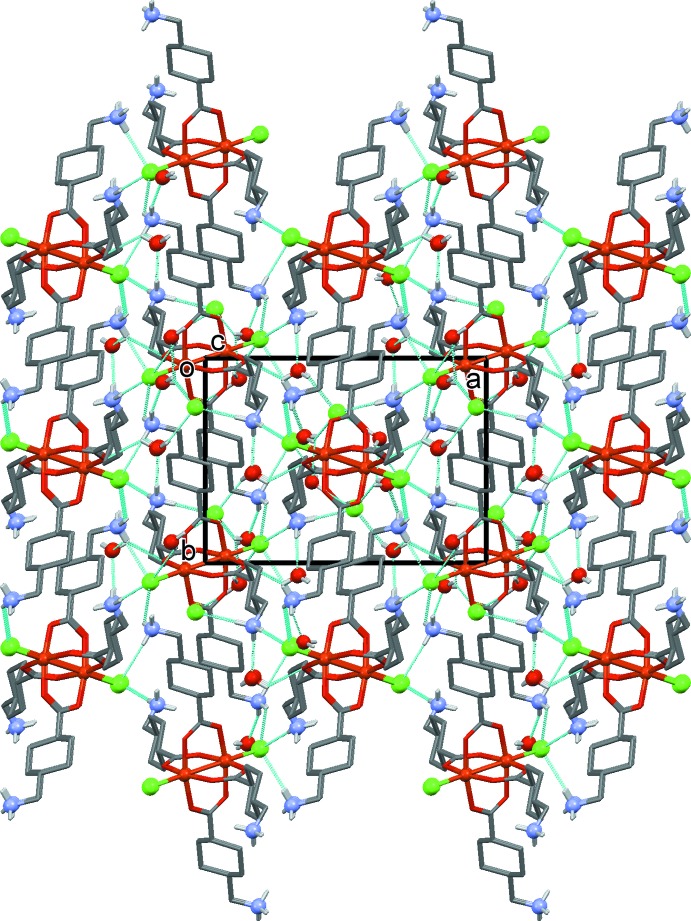
A view along the *c* axis of the crystal structure of the title compound, with the hydrogen bonds shown as dashed lines (see Table 2 ▸). The C-bound H atoms have been omitted for clarity.

In the crystal, the NH_3_^+^ groups of the zwitterionic tranexamic acid units and the coordinating and free Cl^*–*^ ions are linked by a series of N—H⋯Cl hydrogen bonds forming a three-dimensional framework (Table 2 ▸ and Fig. 3 ▸). This framework is strengthened by a series of N—H⋯O_water_, O_water_—H⋯Cl and O_water_—H⋯O hydrogen bonds (Table 2 ▸ and Fig. 4 ▸). The packing index, or percentage of filled space, is 67.1 (Kitajgorodskij, 1973 ▸) and the unit cell contains no residual solvent-accessible voids.

## Database survey   

A search of the Cambridge Structural Database (CSD, Version 5.38, update May 2017; Groom *et al.*, 2016 ▸) for the skeleton of amino-methyl-cyclo­hexane-carb­oxy­lic acid gave 13 hits, of which six structures concern the *cis*- and *trans*-isomers. The crystal structures of the hydro­bromide of the *trans*-isomer (CSD refcode: CHCAHB) and the hydro­chloride of the *cis*-isomer (CHCAHC) were reported in 1966 (Kadoya *et al.*, 1966 ▸). The crystal structure of the hydro­bromide of the *cis*-isomer has also been reported (AMHCAC; Groth & Hassel, 1965 ▸), and that of the free *cis*-isomer (AMCHCA; Yamazaki *et al.*, 1981 ▸), which does not exist as a zwitterion in the solid state. The room temperature analysis of the *trans*-isomer (TA), *viz*. tranexamic acid (AMMCHC10; Groth, 1968 ▸), and a low-temperature analysis at 173 K (AMMCHC11; Shahzadi *et al.*, 2007 ▸) showed that it crystallizes in the chiral ortho­rhom­bic space group *P*2_1_2_1_2_1_ and exists as a zwitterion in the solid state. Inter­estingly, in the low-temperature structure it can be seen that the carboxyl­ate group (COO^−^) is inclined to the mean plane of the four planar atoms of the cyclo­hexane ring by 48.9 (2)°, compared to 67.5 (6) and 85.8 (7)° in the title compound. The plane of the ammonio­methyl unit (C_ar_—C—N) is inclined to the same mean plane of the four planar atoms of the cyclo­hexane ring by 37.4 (2)°, compared to 34.9 (6) and 47.5 (6)° in the title compound. Hence, on complexation the cyclo­hexane rings are rotated about the C_carboxyl­ate_—C_cyclo­hexa­ne_ bonds (C1—C2 and C9—C10), most probably to minimize steric hindrance.

In the CSD over 1500 copper(II) paddle-wheel structures have been deposited. There are only 13 compounds in which the axial position is occupied by a Cl^−^ anion (see *Supporting information*). The Cu⋯Cu distances vary from *ca* 2.63 to 2.84 Å, with the carboxyl­ate groups being inclined to one another by *ca* 84.65–90°, and the Cu—Cl distances varying from *ca* 2.41 to 2.49 Å. The values observed for the title compound fall within these limits (see Section 2, *Structural commentary*). In all 13 compounds the copper atoms have perfect square-pyramidal geometry, with *τ*_5_ = 0.0.

## Synthesis and crystallization   

Tranexamic acid (0.785 g, 0.5 mmol) dissolved in 10 ml of deionized water was added dropwise to a transparent blue solution of CuCl_2_·2H_2_O (0.426 g, 0.25 mmol) in 20 ml of aceto­nitrile at ambient temperature and the mixture was stirred for 30 min. The green solution obtained was filtered and the filtrate kept undisturbed at room temperature for slow evaporation. After five days green plate-like crystals of the title compound were obtained.

## Refinement   

Crystal data, data collection and structure refinement details are summarized in Table 3 ▸. The H atoms of the water mol­ecules were located in difference-Fourier maps and refined with distance restraints: O—H = 0.88 (2) Å with *U*_iso_(H) = 1.5*U*_eq_(O). The ammonium H atoms and the C-bound H atoms were included in calculated positions and treated as riding: N—H = 0.91 Å, C-H = 0.99–1.00 Å with *U*_iso_(H) = 1.5*U*_eq_(N-ammonium) and 1.2*U*_eq_(C) for other H atoms. In the final difference-Fourier map the residual density peaks [Δρ_max_, Δρ_min_ 1.80, −0.93 e Å^−3^] are located at a distance of 1.2 and 0.9 Å, respectively, from the copper atoms.

## Supplementary Material

Crystal structure: contains datablock(s) I, Global. DOI: 10.1107/S2056989017012543/wm5416sup1.cif

Structure factors: contains datablock(s) I. DOI: 10.1107/S2056989017012543/wm5416Isup2.hkl

CSD search of axially Cl- ligated Cu-Cu paddle-wheel structures. DOI: 10.1107/S2056989017012543/wm5416sup3.pdf

CCDC reference: 1571897

Additional supporting information:  crystallographic information; 3D view; checkCIF report

## Figures and Tables

**Table 1:** Selected geometric parameters (Å, °)

Cu1—Cu1^i^	2.6649 (11)	Cu1—O4^i^	1.965 (4)
Cu1—O1	1.950 (4)	Cu1—O3	1.991 (3)
Cu1—O2^i^	1.955 (4)	Cu1—Cl1	2.4990 (12)
			
O1—Cu1—O2^i^	167.02 (15)	O4^i^—Cu1—Cl1	92.57 (11)
O1—Cu1—O4^i^	89.3 (2)	O3—Cu1—Cl1	100.08 (10)
O2^i^—Cu1—O4^i^	89.9 (2)	O1—Cu1—Cu1^i^	83.04 (11)
O1—Cu1—O3	88.33 (18)	O2^i^—Cu1—Cu1^i^	84.05 (11)
O2^i^—Cu1—O3	89.60 (18)	O4^i^—Cu1—Cu1^i^	80.24 (11)
O4^i^—Cu1—O3	167.27 (15)	O3—Cu1—Cu1^i^	87.06 (10)
O1—Cu1—Cl1	94.31 (11)	Cl1—Cu1—Cu1^i^	172.34 (4)
O2^i^—Cu1—Cl1	98.68 (11)		

Symmetry code: (i) 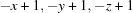.

**Table 2:** Hydrogen-bond geometry (Å, °)

*D*—H⋯*A*	*D*—H	H⋯*A*	*D*⋯*A*	*D*—H⋯*A*
N1—H1*A*⋯Cl1^ii^	0.91	2.63	3.316 (5)	133
N1—H1*C*⋯Cl1^iii^	0.91	2.52	3.394 (5)	160
N2—H2*B*⋯Cl1^iv^	0.91	2.25	3.123 (5)	160
N2—H2*C*⋯Cl2^v^	0.91	2.24	3.151 (6)	179
N1—H1*B*⋯O1*W* ^vi^	0.91	1.90	2.760 (7)	156
N2—H2*A*⋯O2*W* ^vii^	0.91	2.06	2.789 (9)	137
O1*W*—H1*WA*⋯Cl2^v^	0.89 (2)	2.57 (8)	3.218 (6)	130 (8)
O2*W*—H2*WA*⋯Cl2^viii^	0.90 (2)	2.46 (7)	3.176 (6)	137 (9)
O2*W*—H2*WB*⋯O3^i^	0.90 (2)	2.22 (8)	2.820 (6)	124 (8)
O3*W*—H3*WA*⋯Cl1^ix^	0.88 (2)	2.66 (6)	3.258 (5)	127 (6)
O3*W*—H3*WB*⋯Cl2^x^	0.88 (2)	2.35 (3)	3.182 (5)	158 (7)

Symmetry codes: (i) 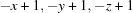; (ii) 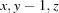; (iii) 
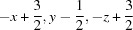
; (iv) 
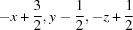
; (v) 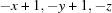; (vi) 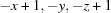; (vii) 
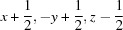
; (viii) 
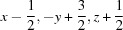
; (ix) 
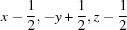
; (x) 
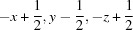
.

**Table 3:** Experimental details

Crystal data	
Chemical formula	[Cu_2_Cl_2_(C_8_H_15_NO_2_)_4_](Cl_2_)·6H_2_O
*M*_r_	1005.81
Crystal system, space group	Monoclinic, *P*2_1_/*n*
Temperature (K)	153
*a*, *b*, *c* (Å)	14.7100 (11), 10.7163 (6), 14.9312 (12)
β (°)	91.828 (10)
*V* (Å^3^)	2352.5 (3)
*Z*	2
Radiation type	Mo *K*α
μ (mm^−1^)	1.19
Crystal size (mm)	0.34 × 0.30 × 0.20

Data collection	
Diffractometer	STOE *IPDS* 1
Absorption correction	Multi-scan (*MULABS*; Spek, 2009 ▸)
*T*_min_, *T*_max_	0.712, 1.000
No. of measured, independent and observed [*I* > 2σ(*I*)] reflections	18018, 4545, 3172
*R*_int_	0.091
(sin θ/λ)_max_ (Å^−1^)	0.615

Refinement	
*R*[*F* ^2^ > 2σ(*F* ^2^)], *wR*(*F* ^2^), *S*	0.067, 0.196, 1.02
No. of reflections	4545
No. of parameters	273
No. of restraints	9
H-atom treatment	H atoms treated by a mixture of independent and constrained refinement
Δρ_max_, Δρ_min_ (e Å^−3^)	1.80, −0.93

Computer programs: *EXPOSE*, *CELL* and *INTEGRATE* in *IPDS-I* (Stoe & Cie, 2004 ▸), *SHELXS2016/6* (Sheldrick, 2008 ▸), *Mercury* (Macrae *et al.*, 2008 ▸), *SHELXL2016/6* (Sheldrick, 2015 ▸), *PLATON* (Spek, 2009 ▸) and *publCIF* (Westrip, 2010 ▸).

## supplementary crystallographic information

### Crystal data

**Table:**

[Cu_2_Cl_2_(C_8_H_15_NO_2_)_4_](Cl_2_)·6H_2_O	*F*(000) = 1060
*M**_r_* = 1005.81	*D*_x_ = 1.420 Mg m^−^^3^
Monoclinic, *P*2_1_/*n*	Mo *K*α radiation, λ = 0.71073 Å
*a* = 14.7100 (11) Å	Cell parameters from 8000 reflections
*b* = 10.7163 (6) Å	θ = 2.3–25.9°
*c* = 14.9312 (12) Å	µ = 1.19 mm^−^^1^
β = 91.828 (10)°	*T* = 153 K
*V* = 2352.5 (3) Å^3^	Plate, green
*Z* = 2	0.34 × 0.30 × 0.20 mm

### Data collection

**Table:**

STOE IPDS 1 diffractometer	4545 independent reflections
Radiation source: fine-focus sealed tube	3172 reflections with *I* > 2σ(*I*)
Plane graphite monochromator	*R*_int_ = 0.091
φ rotation scans	θ_max_ = 25.9°, θ_min_ = 2.3°
Absorption correction: multi-scan (MULABS; Spek, 2009)	*h* = −18→18
*T*_min_ = 0.712, *T*_max_ = 1.000	*k* = −13→13
18018 measured reflections	*l* = −18→18

### Refinement

**Table:**

Refinement on *F*^2^	Primary atom site location: structure-invariant direct methods
Least-squares matrix: full	Secondary atom site location: difference Fourier map
*R*[*F*^2^ > 2σ(*F*^2^)] = 0.067	Hydrogen site location: mixed
*wR*(*F*^2^) = 0.196	H atoms treated by a mixture of independent and constrained refinement
*S* = 1.02	*w* = 1/[σ^2^(*F*_o_^2^) + (0.1331*P*)^2^] where *P* = (*F*_o_^2^ + 2*F*_c_^2^)/3
4545 reflections	(Δ/σ)_max_ < 0.001
273 parameters	Δρ_max_ = 1.80 e Å^−^^3^
9 restraints	Δρ_min_ = −0.93 e Å^−^^3^

### Special details

**Table:**

Geometry. All esds (except the esd in the dihedral angle between two l.s. planes) are estimated using the full covariance matrix. The cell esds are taken into account individually in the estimation of esds in distances, angles and torsion angles; correlations between esds in cell parameters are only used when they are defined by crystal symmetry. An approximate (isotropic) treatment of cell esds is used for estimating esds involving l.s. planes.

### Fractional atomic coordinates and isotropic or equivalent isotropic displacement parameters (Å^2^)

**Table:**

	*x*	*y*	*z*	*U*_iso_*/*U*_eq_	
Cu1	0.57200 (3)	0.53341 (6)	0.55087 (4)	0.0195 (2)	
Cl1	0.69386 (7)	0.59126 (13)	0.66374 (7)	0.0253 (3)	
O1	0.5727 (3)	0.3572 (4)	0.5833 (3)	0.0384 (10)	
O2	0.4505 (3)	0.3014 (4)	0.5009 (3)	0.0369 (10)	
O3	0.6477 (2)	0.4905 (4)	0.4471 (2)	0.0297 (9)	
O4	0.5250 (2)	0.4381 (5)	0.3640 (3)	0.0445 (12)	
N1	0.6876 (3)	−0.1791 (5)	0.8123 (3)	0.0339 (11)	
H1A	0.715646	−0.207468	0.762784	0.051*	
H1B	0.688742	−0.239545	0.855069	0.051*	
H1C	0.717108	−0.110116	0.833690	0.051*	
N2	0.6727 (3)	0.1961 (6)	−0.0237 (3)	0.0451 (14)	
H2A	0.674668	0.130093	0.014673	0.068*	
H2B	0.702133	0.175997	−0.074399	0.068*	
H2C	0.613684	0.215079	−0.038025	0.068*	
C1	0.5154 (3)	0.2771 (5)	0.5551 (3)	0.0244 (11)	
C2	0.5225 (4)	0.1471 (5)	0.5916 (4)	0.0299 (12)	
H2	0.490146	0.089283	0.548741	0.036*	
C3	0.6224 (4)	0.1033 (6)	0.6034 (4)	0.0377 (14)	
H3A	0.650187	0.098871	0.543974	0.045*	
H3B	0.657016	0.165255	0.640008	0.045*	
C4	0.6295 (4)	−0.0230 (6)	0.6482 (4)	0.0388 (14)	
H4A	0.694444	−0.045681	0.657197	0.047*	
H4B	0.600371	−0.086743	0.608849	0.047*	
C5	0.5841 (3)	−0.0227 (5)	0.7379 (4)	0.0288 (12)	
H5	0.614942	0.042295	0.776158	0.035*	
C6	0.4837 (4)	0.0149 (6)	0.7258 (6)	0.0472 (18)	
H6A	0.451049	−0.048267	0.688621	0.057*	
H6B	0.455311	0.017746	0.784995	0.057*	
C7	0.4754 (3)	0.1418 (6)	0.6811 (4)	0.0372 (14)	
H7A	0.502404	0.205796	0.721666	0.045*	
H7B	0.410239	0.162109	0.671203	0.045*	
C8	0.5915 (4)	−0.1465 (6)	0.7881 (4)	0.0397 (14)	
H8A	0.556328	−0.141231	0.843406	0.048*	
H8B	0.564509	−0.213527	0.750187	0.048*	
C9	0.6099 (3)	0.4514 (5)	0.3760 (3)	0.0244 (11)	
C10	0.6679 (3)	0.4182 (5)	0.2966 (3)	0.0244 (11)	
H10	0.733515	0.427604	0.315003	0.029*	
C11	0.6461 (4)	0.5081 (6)	0.2187 (4)	0.0288 (12)	
H11A	0.579838	0.507245	0.204844	0.035*	
H11B	0.663545	0.593965	0.236560	0.035*	
C12	0.6970 (4)	0.4710 (6)	0.1351 (3)	0.0299 (12)	
H12A	0.681611	0.530032	0.085930	0.036*	
H12B	0.763338	0.475985	0.147985	0.036*	
C13	0.6722 (3)	0.3405 (5)	0.1063 (3)	0.0262 (12)	
H13	0.604822	0.337427	0.095228	0.031*	
C14	0.6965 (4)	0.2495 (6)	0.1830 (4)	0.0332 (13)	
H14A	0.677871	0.164112	0.164965	0.040*	
H14B	0.763281	0.249287	0.193720	0.040*	
C15	0.6507 (4)	0.2838 (6)	0.2693 (4)	0.0319 (12)	
H15A	0.584401	0.270120	0.261501	0.038*	
H15B	0.673527	0.227990	0.317868	0.038*	
C16	0.7174 (4)	0.3051 (6)	0.0196 (4)	0.0347 (14)	
H16A	0.714618	0.376924	−0.022083	0.042*	
H16B	0.782239	0.285487	0.032601	0.042*	
Cl2	0.53120 (11)	0.73439 (19)	0.07190 (12)	0.0537 (5)	
O1W	0.3515 (3)	0.3850 (6)	0.0849 (3)	0.0600 (14)	
H1WA	0.385 (4)	0.405 (9)	0.038 (3)	0.090*	
H1WB	0.393 (4)	0.383 (9)	0.130 (3)	0.090*	
O2W	0.1694 (4)	0.5612 (6)	0.5040 (4)	0.0651 (15)	
H2WA	0.134 (5)	0.589 (9)	0.548 (5)	0.098*	
H2WB	0.222 (3)	0.602 (8)	0.514 (6)	0.098*	
O3W	0.1194 (3)	0.1039 (6)	0.3118 (3)	0.0619 (15)	
H3WA	0.104 (5)	0.075 (8)	0.259 (3)	0.093*	
H3WB	0.073 (4)	0.148 (8)	0.329 (5)	0.093*	

### Atomic displacement parameters (Å^2^)

**Table:**

	*U*^11^	*U*^22^	*U*^33^	*U*^12^	*U*^13^	*U*^23^
Cu1	0.0152 (3)	0.0291 (4)	0.0144 (3)	0.0005 (2)	0.0036 (2)	0.0010 (2)
Cl1	0.0213 (6)	0.0352 (8)	0.0193 (6)	−0.0045 (5)	0.0001 (4)	0.0005 (5)
O1	0.035 (2)	0.039 (3)	0.041 (2)	−0.0092 (18)	−0.0129 (17)	0.0117 (19)
O2	0.035 (2)	0.033 (2)	0.041 (2)	0.0018 (17)	−0.0158 (17)	−0.0035 (19)
O3	0.0195 (16)	0.050 (3)	0.0195 (17)	−0.0029 (16)	0.0022 (13)	−0.0049 (17)
O4	0.0187 (18)	0.088 (4)	0.027 (2)	−0.0061 (19)	0.0057 (14)	−0.018 (2)
N1	0.031 (2)	0.032 (3)	0.038 (3)	0.007 (2)	−0.0064 (19)	0.001 (2)
N2	0.041 (3)	0.056 (4)	0.039 (3)	0.001 (3)	0.018 (2)	−0.019 (3)
C1	0.024 (2)	0.031 (3)	0.018 (2)	0.004 (2)	0.0067 (19)	0.004 (2)
C2	0.032 (3)	0.025 (3)	0.032 (3)	0.001 (2)	−0.008 (2)	−0.003 (2)
C3	0.032 (3)	0.048 (4)	0.034 (3)	0.015 (3)	0.016 (2)	0.011 (3)
C4	0.039 (3)	0.040 (4)	0.038 (3)	0.015 (3)	−0.001 (2)	0.003 (3)
C5	0.020 (2)	0.029 (3)	0.037 (3)	0.001 (2)	−0.002 (2)	0.004 (2)
C6	0.022 (3)	0.036 (4)	0.084 (5)	0.002 (2)	0.010 (3)	0.022 (4)
C7	0.019 (2)	0.037 (4)	0.056 (4)	0.007 (2)	0.010 (2)	0.019 (3)
C8	0.028 (3)	0.040 (4)	0.051 (4)	0.005 (3)	0.004 (2)	0.009 (3)
C9	0.022 (2)	0.030 (3)	0.022 (2)	0.001 (2)	0.0045 (19)	0.004 (2)
C10	0.014 (2)	0.039 (3)	0.021 (2)	0.001 (2)	0.0046 (17)	0.000 (2)
C11	0.034 (3)	0.029 (3)	0.024 (3)	0.002 (2)	0.008 (2)	−0.001 (2)
C12	0.032 (3)	0.037 (4)	0.021 (3)	0.004 (2)	0.010 (2)	0.004 (2)
C13	0.020 (2)	0.035 (3)	0.024 (2)	0.004 (2)	0.0101 (18)	−0.006 (2)
C14	0.031 (3)	0.030 (3)	0.039 (3)	0.002 (2)	0.011 (2)	0.000 (3)
C15	0.036 (3)	0.031 (3)	0.030 (3)	0.003 (2)	0.011 (2)	0.002 (2)
C16	0.029 (3)	0.047 (4)	0.028 (3)	0.003 (2)	0.013 (2)	−0.012 (3)
Cl2	0.0453 (9)	0.0647 (13)	0.0518 (10)	−0.0012 (8)	0.0122 (7)	0.0128 (9)
O1W	0.062 (3)	0.068 (4)	0.051 (3)	0.008 (3)	0.014 (2)	0.021 (3)
O2W	0.048 (3)	0.075 (4)	0.071 (4)	0.012 (3)	−0.008 (3)	−0.019 (3)
O3W	0.052 (3)	0.081 (4)	0.053 (3)	0.018 (3)	0.000 (2)	−0.034 (3)

### Geometric parameters (Å, º)

**Table:**

Cu1—Cu1^i^	2.6649 (11)	C6—H6A	0.9900
Cu1—O1	1.950 (4)	C6—H6B	0.9900
Cu1—O2^i^	1.955 (4)	C7—H7A	0.9900
Cu1—O4^i^	1.965 (4)	C7—H7B	0.9900
Cu1—O3	1.991 (3)	C8—H8A	0.9900
Cu1—Cl1	2.4990 (12)	C8—H8B	0.9900
O1—C1	1.265 (7)	C9—C10	1.526 (6)
O2—C1	1.258 (6)	C10—C15	1.516 (8)
O3—C9	1.255 (6)	C10—C11	1.536 (7)
O4—C9	1.264 (6)	C10—H10	1.0000
N1—C8	1.489 (7)	C11—C12	1.527 (7)
N1—H1A	0.9100	C11—H11A	0.9900
N1—H1B	0.9100	C11—H11B	0.9900
N1—H1C	0.9100	C12—C13	1.505 (8)
N2—C16	1.479 (8)	C12—H12A	0.9900
N2—H2A	0.9100	C12—H12B	0.9900
N2—H2B	0.9100	C13—C16	1.522 (7)
N2—H2C	0.9100	C13—C14	1.538 (8)
C1—C2	1.499 (8)	C13—H13	1.0000
C2—C7	1.526 (8)	C14—C15	1.518 (7)
C2—C3	1.547 (7)	C14—H14A	0.9900
C2—H2	1.0000	C14—H14B	0.9900
C3—C4	1.512 (9)	C15—H15A	0.9900
C3—H3A	0.9900	C15—H15B	0.9900
C3—H3B	0.9900	C16—H16A	0.9900
C4—C5	1.515 (8)	C16—H16B	0.9900
C4—H4A	0.9900	O1W—H1WA	0.89 (2)
C4—H4B	0.9900	O1W—H1WB	0.90 (2)
C5—C8	1.526 (8)	O2W—H2WA	0.90 (2)
C5—C6	1.536 (7)	O2W—H2WB	0.90 (2)
C5—H5	1.0000	O3W—H3WA	0.88 (2)
C6—C7	1.518 (9)	O3W—H3WB	0.88 (2)
			
O1—Cu1—O2^i^	167.02 (15)	C5—C6—H6B	109.6
O1—Cu1—O4^i^	89.3 (2)	H6A—C6—H6B	108.1
O2^i^—Cu1—O4^i^	89.9 (2)	C6—C7—C2	112.7 (5)
O1—Cu1—O3	88.33 (18)	C6—C7—H7A	109.0
O2^i^—Cu1—O3	89.60 (18)	C2—C7—H7A	109.0
O4^i^—Cu1—O3	167.27 (15)	C6—C7—H7B	109.0
O1—Cu1—Cl1	94.31 (11)	C2—C7—H7B	109.0
O2^i^—Cu1—Cl1	98.68 (11)	H7A—C7—H7B	107.8
O4^i^—Cu1—Cl1	92.57 (11)	N1—C8—C5	112.0 (5)
O3—Cu1—Cl1	100.08 (10)	N1—C8—H8A	109.2
O1—Cu1—Cu1^i^	83.04 (11)	C5—C8—H8A	109.2
O2^i^—Cu1—Cu1^i^	84.05 (11)	N1—C8—H8B	109.2
O4^i^—Cu1—Cu1^i^	80.24 (11)	C5—C8—H8B	109.2
O3—Cu1—Cu1^i^	87.06 (10)	H8A—C8—H8B	107.9
Cl1—Cu1—Cu1^i^	172.34 (4)	O3—C9—O4	124.6 (5)
C1—O1—Cu1	125.2 (3)	O3—C9—C10	119.4 (4)
C1—O2—Cu1^i^	123.8 (4)	O4—C9—C10	116.0 (4)
C9—O3—Cu1	119.4 (3)	C15—C10—C9	109.8 (4)
C9—O4—Cu1^i^	128.7 (3)	C15—C10—C11	111.3 (4)
C8—N1—H1A	109.5	C9—C10—C11	109.5 (4)
C8—N1—H1B	109.5	C15—C10—H10	108.7
H1A—N1—H1B	109.5	C9—C10—H10	108.7
C8—N1—H1C	109.5	C11—C10—H10	108.7
H1A—N1—H1C	109.5	C12—C11—C10	111.1 (4)
H1B—N1—H1C	109.5	C12—C11—H11A	109.4
C16—N2—H2A	109.5	C10—C11—H11A	109.4
C16—N2—H2B	109.5	C12—C11—H11B	109.4
H2A—N2—H2B	109.5	C10—C11—H11B	109.4
C16—N2—H2C	109.5	H11A—C11—H11B	108.0
H2A—N2—H2C	109.5	C13—C12—C11	110.8 (4)
H2B—N2—H2C	109.5	C13—C12—H12A	109.5
O2—C1—O1	123.9 (5)	C11—C12—H12A	109.5
O2—C1—C2	118.0 (5)	C13—C12—H12B	109.5
O1—C1—C2	118.1 (4)	C11—C12—H12B	109.5
C1—C2—C7	108.9 (5)	H12A—C12—H12B	108.1
C1—C2—C3	112.2 (5)	C12—C13—C16	111.5 (5)
C7—C2—C3	110.2 (4)	C12—C13—C14	109.2 (4)
C1—C2—H2	108.5	C16—C13—C14	112.2 (5)
C7—C2—H2	108.5	C12—C13—H13	107.9
C3—C2—H2	108.5	C16—C13—H13	107.9
C4—C3—C2	112.0 (5)	C14—C13—H13	107.9
C4—C3—H3A	109.2	C15—C14—C13	112.3 (5)
C2—C3—H3A	109.2	C15—C14—H14A	109.1
C4—C3—H3B	109.2	C13—C14—H14A	109.1
C2—C3—H3B	109.2	C15—C14—H14B	109.1
H3A—C3—H3B	107.9	C13—C14—H14B	109.1
C3—C4—C5	111.3 (5)	H14A—C14—H14B	107.9
C3—C4—H4A	109.4	C10—C15—C14	112.5 (5)
C5—C4—H4A	109.4	C10—C15—H15A	109.1
C3—C4—H4B	109.4	C14—C15—H15A	109.1
C5—C4—H4B	109.4	C10—C15—H15B	109.1
H4A—C4—H4B	108.0	C14—C15—H15B	109.1
C4—C5—C8	113.9 (5)	H15A—C15—H15B	107.8
C4—C5—C6	110.2 (5)	N2—C16—C13	111.6 (5)
C8—C5—C6	109.8 (5)	N2—C16—H16A	109.3
C4—C5—H5	107.5	C13—C16—H16A	109.3
C8—C5—H5	107.5	N2—C16—H16B	109.3
C6—C5—H5	107.5	C13—C16—H16B	109.3
C7—C6—C5	110.5 (5)	H16A—C16—H16B	108.0
C7—C6—H6A	109.6	H1WA—O1W—H1WB	102 (3)
C5—C6—H6A	109.6	H2WA—O2W—H2WB	103 (3)
C7—C6—H6B	109.6	H3WA—O3W—H3WB	106 (3)
			
Cu1^i^—O2—C1—O1	1.1 (7)	Cu1—O3—C9—O4	1.0 (8)
Cu1^i^—O2—C1—C2	178.1 (4)	Cu1—O3—C9—C10	−179.8 (4)
Cu1—O1—C1—O2	0.9 (7)	Cu1^i^—O4—C9—O3	−2.3 (9)
Cu1—O1—C1—C2	−176.1 (4)	Cu1^i^—O4—C9—C10	178.5 (4)
O2—C1—C2—C7	−94.2 (6)	O3—C9—C10—C15	122.8 (5)
O1—C1—C2—C7	82.9 (6)	O4—C9—C10—C15	−57.9 (6)
O2—C1—C2—C3	143.5 (5)	O3—C9—C10—C11	−114.7 (5)
O1—C1—C2—C3	−39.4 (7)	O4—C9—C10—C11	64.5 (6)
C1—C2—C3—C4	174.7 (5)	C15—C10—C11—C12	−53.7 (6)
C7—C2—C3—C4	53.1 (7)	C9—C10—C11—C12	−175.2 (4)
C2—C3—C4—C5	−56.3 (7)	C10—C11—C12—C13	58.9 (6)
C3—C4—C5—C8	−178.1 (5)	C11—C12—C13—C16	176.2 (4)
C3—C4—C5—C6	57.9 (7)	C11—C12—C13—C14	−59.3 (5)
C4—C5—C6—C7	−57.2 (8)	C12—C13—C14—C15	56.5 (6)
C8—C5—C6—C7	176.5 (6)	C16—C13—C14—C15	−179.4 (5)
C5—C6—C7—C2	55.9 (8)	C9—C10—C15—C14	172.3 (4)
C1—C2—C7—C6	−176.8 (5)	C11—C10—C15—C14	50.9 (6)
C3—C2—C7—C6	−53.3 (7)	C13—C14—C15—C10	−52.9 (6)
C4—C5—C8—N1	63.3 (7)	C12—C13—C16—N2	−162.4 (5)
C6—C5—C8—N1	−172.5 (5)	C14—C13—C16—N2	74.9 (6)

Symmetry code: (i) −*x*+1, −*y*+1, −*z*+1.

### Hydrogen-bond geometry (Å, º)

**Table:**

*D*—H···*A*	*D*—H	H···*A*	*D*···*A*	*D*—H···*A*
N1—H1*A*···Cl1^ii^	0.91	2.63	3.316 (5)	133
N1—H1*C*···Cl1^iii^	0.91	2.52	3.394 (5)	160
N2—H2*B*···Cl1^iv^	0.91	2.25	3.123 (5)	160
N2—H2*C*···Cl2^v^	0.91	2.24	3.151 (6)	179
N1—H1*B*···O1*W*^vi^	0.91	1.90	2.760 (7)	156
N2—H2*A*···O2*W*^vii^	0.91	2.06	2.789 (9)	137
O1*W*—H1*WA*···Cl2^v^	0.89 (2)	2.57 (8)	3.218 (6)	130 (8)
O2*W*—H2*WA*···Cl2^viii^	0.90 (2)	2.46 (7)	3.176 (6)	137 (9)
O2*W*—H2*WB*···O3^i^	0.90 (2)	2.22 (8)	2.820 (6)	124 (8)
O3*W*—H3*WA*···Cl1^ix^	0.88 (2)	2.66 (6)	3.258 (5)	127 (6)
O3*W*—H3*WB*···Cl2^x^	0.88 (2)	2.35 (3)	3.182 (5)	158 (7)

Symmetry codes: (i) −*x*+1, −*y*+1, −*z*+1; (ii) *x*, *y*−1, *z*; (iii) −*x*+3/2, *y*−1/2, −*z*+3/2; (iv) −*x*+3/2, *y*−1/2, −*z*+1/2; (v) −*x*+1, −*y*+1, −*z*; (vi) −*x*+1, −*y*, −*z*+1; (vii) *x*+1/2, −*y*+1/2, *z*−1/2; (viii) *x*−1/2, −*y*+3/2, *z*+1/2; (ix) *x*−1/2, −*y*+1/2, *z*−1/2; (x) −*x*+1/2, *y*−1/2, −*z*+1/2.
