# Supplementary material for: A copper(II) paddle-wheel structure of tranexamic acid: di­chloro-tetra­kis­[μ-4-(ammonio­meth­yl)cyclo­hexane-1-carboxyl­ato-O,O′]dicopper(II) dichloride hexa­hydrate
Source: Acta Crystallogr E Crystallogr Commun. 2017 Sep 8;73(Pt 10):1421–5. doi: 10.1107/S2056989017012543 (PMC5730287; doi:10.1107/S2056989017012543)

Search Overview

Search:	search5
Date/Time done:	Sun Aug 20 15:58:38 2017
Database(s):	CSD version 5.38 updates (Nov 2016)<br>CSD version 5.38 (November 2016)<br>CSD version 5.38 (November 2016)<br>CSD version 5.38 updates (Feb 2017)<br>CSD version 5.38 updates (May 2017)
Restriction Info:	No refcode restrictions applied
Filters:	None
Percentage Completed:	100%
Number of Hits:	17

Single query used. Search found structures that:

match

Query 1

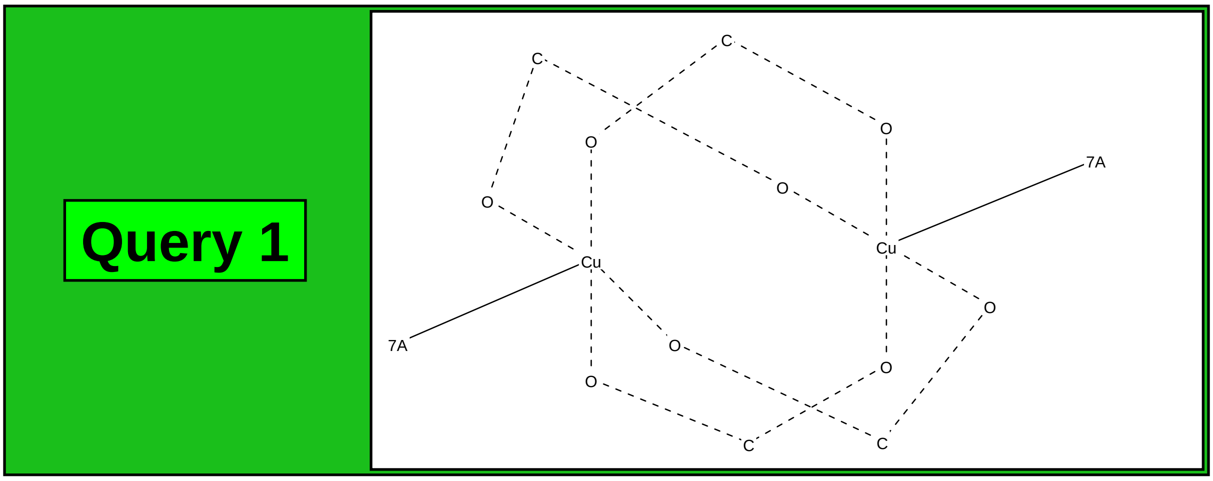

Search: search5 (Sun Aug 20 15:58:38 2017): Hits 1-4

FIWGAT

Reference: H.Nakagawa, Y.Kani, M.Tsuchimoto, S.Ohba, M.Hideaki, T.Tokii (1999) *Acta Crystallogr.,Sect.C:Cryst.Struct.Commun.* ,**55,**882

Formula: $C_{28} H_{28} Cl_2 Cu_2 N_4 O_8{}^{2+},2(Cl_1{}^{1-}),8(H_2 O_1)$

Compound Name: tetrakis(μ_2-N-Methylpyridinium-3-carboxylate)-dichloro-di-copper(ii) dichloride octahydrate

Synonym: tetrakis(μ_2-Trigonelline-O,O')-dichloro-di-copper(ii) dichloride octahydrate

Space Group:	P-1	***Cell:***	***a*** 10.156(2)	***b*** 13.883(2)	***c*** 7.793(1)
Space Group No.:	2	***(Å,°)***	α 99.40(1)	β 109.29(1)	γ 75.57(1)

R-Factor (%): 4.30 ***Temperature(K):*** 295 ***Density(g/cm³):*** 1.596

Parameters

Fragment 1

**DIST1 (D)**	2.770
**DIST2 (D)**	2.413
**DIST3 (D)**	1.985
**ANG1 (X)**	0.028

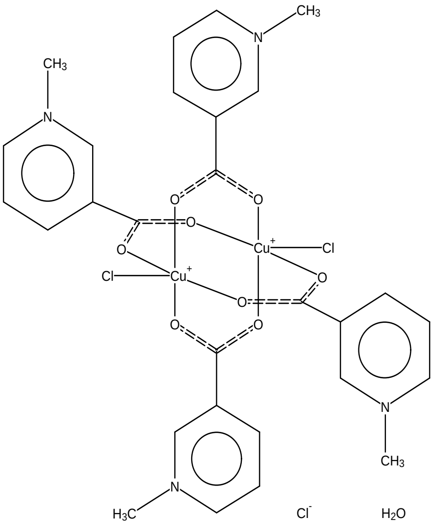

GOLSIK

Reference: M.Shahid, M.Mazhar, M.Zeller, A.D.Hunter (2009) *Acta Crystallogr.,Sect.E:Struct.Rep.Online* ,**65,**m345

Formula: $4(C_5 H_{14} N_1 O_1{}^{1+}),C_8 H_{12} Cl_2 Cu_2 O_8{}^{2-},2(Cl_1{}^{1-})$

Compound Name: tetrakis((3-hydroxypropyl)dimethylammonium) tetrakis(μ_2-acetato)-dichlorido-di-copper(ii) dichloride

Space Group:	P21/c	***Cell:***	***a*** 11.438(3)	***b*** 11.266(3)	***c*** 16.876(4)
Space Group No.:	14	***(Å,°)***	α 90.00	β 97.94(0)	γ 90.00

R-Factor (%): 3.61 ***Temperature(K):*** 100 ***Density(g/cm³):*** 1.421

Parameters

Fragment 1

**DIST1 (D)**	2.679
**DIST2 (D)**	2.480
**DIST3 (D)**	1.976
**ANG1 (X)**	0.000

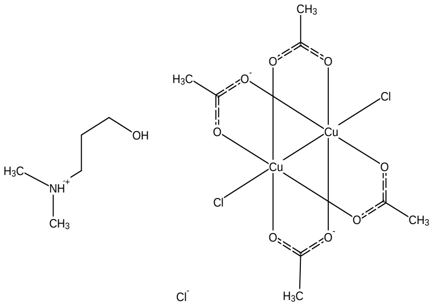

HEMPAQ

Reference: J.Jezierska, T.Glowiak, A.Ozarowski, Y.V.Yablokov, Z.Rzaczynska (1998) *Inorg.Chim.Acta* ,**275,**28

Formula: $C_{12} H_{28} Cl_2 Cu_2 N_4 O_8{}^{2+},2(Cl_1{}^{1-}),H_2 O_1$

Compound Name: tetrakis(μ_2-β-Alanine)-dichloro-di-copper(ii) dichloride monohydrate

Space Group:	P-1	***Cell:***	***a*** 9.444(2)	***b*** 9.498(2)	***c*** 14.107(3)
Space Group No.:	2	***(Å,°)***	α 104.04(3)	β 105.90(3)	γ 97.15(3)

R-Factor (%): 3.41 ***Temperature(K):*** 295 ***Density(g/cm³):*** 1.849

Parameters

Fragment 1

**DIST1 (D)**	2.633
**DIST2 (D)**	2.451
**DIST3 (D)**	1.969
**ANG1 (X)**	87.209

Fragment 2

**DIST1 (D)**	2.661
**DIST2 (D)**	2.439
**DIST3 (D)**	1.975
**ANG1 (X)**	83.652

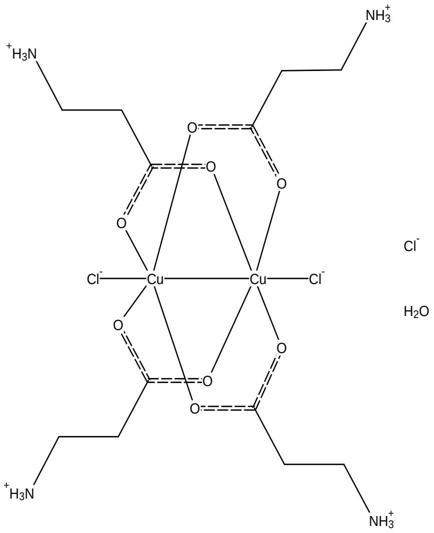

HIYSIR

Reference: Xiao-Ming Chen, Xiao-Long Feng, Zhi-Tao Xu, Xiao-Hong Zhang, Feng Xue, T.C.W.Mak (1998) *Polyhedron* ,**17,**2639

Formula: $C_{32} H_{36} Cl_2 Cu_2 N_4 O_8{}^{2+},2(Cl_1 O_4{}^{1-})$

Compound Name: tetrakis(μ_2-Pyridiniopropionato)-dichloro-di-copper(ii) diperchlorate

Space Group:	Pbca	***Cell:***	***a*** 15.319(3)	***b*** 13.415(2)	***c*** 19.391(2)
Space Group No.:	61	***(Å,°)***	α 90.00	β 90.00	γ 90.00

R-Factor (%): 5.10 ***Temperature(K):*** 295 ***Density(g/cm³):*** 1.669

Parameters

Fragment 1

**DIST1 (D)**	2.762
**DIST2 (D)**	2.453
**DIST3 (D)**	1.974
**ANG1 (X)**	0.028

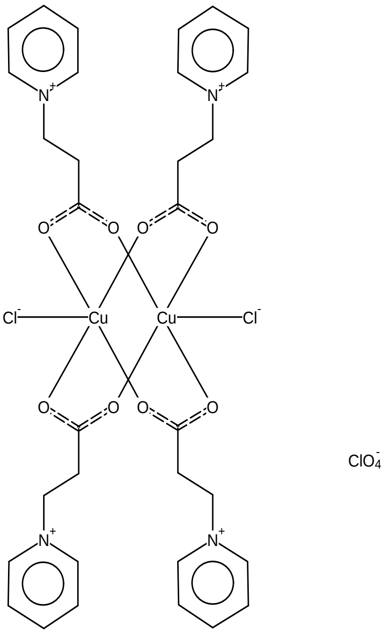

Search: search5 (Sun Aug 20 15:58:38 2017): Hits 5-8

IHAQAJ

Reference: M.R.Silva, J.A.Paixao, A.M.Beja, L.A.da Veiga, J.Martin-Gil (2001) *J.Chem.Cryst.* ,**31,**167

Formula: $C_{20} H_{44} Cl_2 Cu_2 N_4 O_8 {}^{2+}$,2($Cl_1 {}^{1-}$),4($H_2 O_1$)

Compound Name: tetrakis(μ_2-Trimethylammonioacetato-O,O')-dichloro-di-copper(ii) dichloride tetrahydrate

Synonym: tetrakis(μ_2-Betaine-O,O')-dichloro-di-copper(ii) dichloride tetrahydrate

Space Group:	P21/c	***Cell:***	***a*** 11.051(1)	***b*** 14.714(4)	***c*** 11.162(1)
Space Group No.:	14	***(Å,°)***	α 90.00	β 107.40(2)	γ 90.00

R-Factor (%): 3.03 ***Temperature(K):*** 293 ***Density(g/cm^3):*** 1.552

Parameters

Fragment 1	
**DIST1 (D)**	2.766
**DIST2 (D)**	2.418
**DIST3 (D)**	1.968
**ANG1 (X)**	86.249

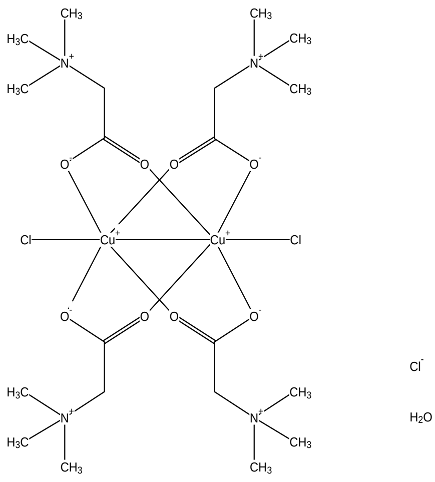

MENQUR

Reference: H.Ackermann, B.Neumuller, K.Dehnicke (2000) *Z.Anorg.Allg.Chem.* ,**626,**1712

Formula: 2($C_8 H_{19} N_1 O_1 P_1 {}^{1+}$),$C_8 H_{12} Cl_2 Cu_2 O_8 {}^{2-}$,4($C_1 H_2 Cl_2$)

Compound Name: bis(N-Acetyltriethylphosphaneiminium) tetrakis(μ_2-acetato)-dichloro-di-copper(ii) dichloromethane solvate

Space Group:	P21/n	***Cell:***	***a*** 7.941(1)	***b*** 23.569(6)	***c*** 13.273(2)
Space Group No.:	14	***(Å,°)***	α 90.00	β 91.00(1)	γ 90.00

R-Factor (%): 5.97 ***Temperature(K):*** 203 ***Density(g/cm^3):*** 1.506

Parameters

Fragment 1	
**DIST1 (D)**	2.687
**DIST2 (D)**	2.465
**DIST3 (D)**	1.977
**ANG1 (X)**	0.000

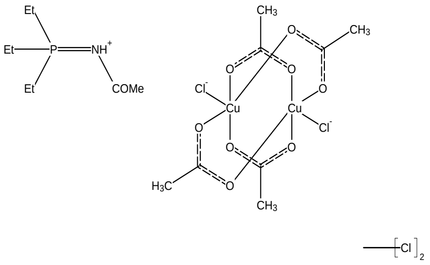

QEWYAT

Reference: L.Wiehl, J.Schreuer, A.Stojic (2006) *Z.Kristallogr.-New Cryst.Struct.* ,**221,**527

Formula: $C_{20} H_{44} Br_2 Cu_2 N_4 O_8 {}^{2+}$,2($Br_1 {}^{1-}$),2($H_2 O_1$)

Compound Name: tetrakis(μ_2-Betaine-O,O')-dibromo-di-copper(ii) dibromide dihydrate

Space Group:	P21/c	***Cell:***	***a*** 11.304(0)	***b*** 14.766(0)	***c*** 11.324(0)
Space Group No.:	14	***(Å,°)***	α 90.00	β 108.19(0)	γ 90.00

R-Factor (%): 3.23 ***Temperature(K):*** 293 ***Density(g/cm^3):*** 1.759

Parameters

Fragment 1	
**DIST1 (D)**	2.768
**DIST2 (D)**	2.556
**DIST3 (D)**	1.977
**ANG1 (X)**	0.034

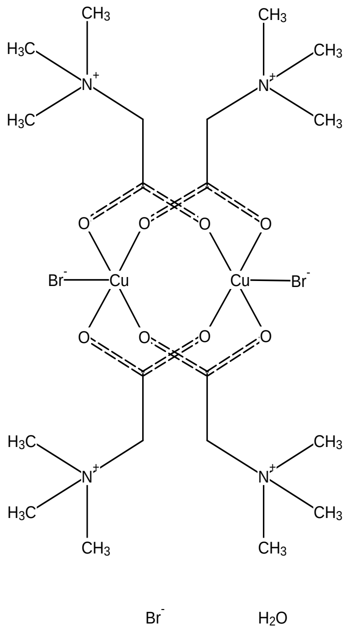

QEWYEX

Reference: J.Schreuer, L.Wiehl, J.Biehler, P.Hofmann (2006) *Z.Kristallogr.-New Cryst.Struct.* ,**221,**529

Formula: $C_{20} H_{44} Br_2 Cu_2 N_4 O_8 {}^{2+}$,$Br_4 Cu_1 {}^{2-}$,$H_2 O_1$

Compound Name: tetrakis(μ_2-Betaine-O,O')-dibromo-di-copper(ii) tetrabromo-copper(ii) monohydrate

Space Group:	P-1	***Cell:***	***a*** 11.681(0)	***b*** 13.285(1)	***c*** 14.719(1)
Space Group No.:	2	***(Å,°)***	α 64.61(0)	β 75.23(0)	γ 74.82(0)

R-Factor (%): 2.95 ***Temperature(K):*** 293 ***Density(g/cm^3):*** 1.955

Parameters

Fragment 1	
**DIST1 (D)**	2.783
**DIST2 (D)**	2.563
**DIST3 (D)**	1.965
**ANG1 (X)**	0.000

Fragment 2	
**DIST1 (D)**	2.820
**DIST2 (D)**	2.529
**DIST3 (D)**	1.981
**ANG1 (X)**	0.000

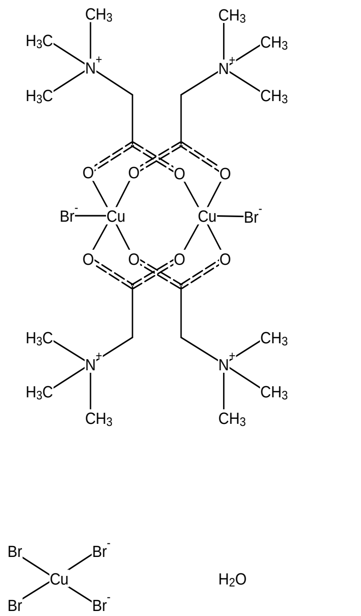

Search: search5 (Sun Aug 20 15:58:38 2017): Hits 9-12

RERTUD

Reference: A.Harada, M.Tsuchimoto, S.Ohba, K.Iwasawa, T.Tokii (1997) *Acta Crystallogr.,Sect.B:Struct.Sci.* ,**53,**654

Formula: $C_{32}H_{20}Cl_2Cu_2O_{12}{}^{2-}$,$2(C_7H_{10}N_1{}^{1+})$,$2(C_6H_6)$

Compound Name: bis(2,3-Dimethylpyridinium) tetrakis(μ_2-benzoylformato-O,O')-dichloro-di-copper(ii) benzene solvate

Space Group: P21/n ***Cell:*** ***a*** 14.332(1) ***b*** 10.855(1) ***c*** 17.719(1)
Space Group No.: 14 ***(Å,°)*** α 90.00 β 91.00(1) γ 90.00

R-Factor (%): 4.76 ***Temperature(K):*** 295 ***Density(g/cm³):*** 1.406

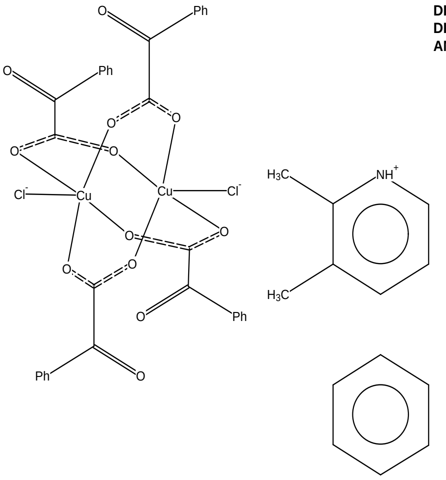

Parameters

Fragment 1
DIST1 (D) 2.843
DIST2 (D) 2.394
DIST3 (D) 1.977
ANG1 (X) 0.000

SAPDET

Reference: N.Lah, I.Leban (2005) *Acta Crystallogr.,Sect.E:Struct.Rep.Online* ,**61,**m835

Formula: $C_{24}H_{52}Cl_2Cu_2N_4O_8{}^{2+}$,$2(Cl_1{}^{1-})$,$4(H_2O_1)$

Compound Name: tetrakis(μ_2-6-Ammoniohexanoato-O,O')-bis(chloro-copper(ii)) dichloride tetrahydrate

Space Group: P21/n ***Cell:*** ***a*** 9.412(0) ***b*** 12.185(0) ***c*** 17.585(0)
Space Group No.: 14 ***(Å,°)*** α 90.00 β 105.44(0) γ 90.00

R-Factor (%): 3.60 ***Temperature(K):*** 150 ***Density(g/cm³):*** 1.479

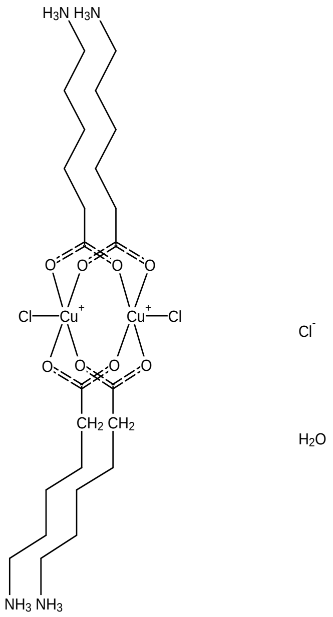

Parameters

Fragment 1
DIST1 (D) 2.651
DIST2 (D) 2.486
DIST3 (D) 1.978
ANG1 (X) 0.000

SASVOY

Reference: M.R.Silva, A.M.Beja, J.A.Paixao, J.Martin-Gil (2005) *Acta Crystallogr.,Sect.C:Cryst.Struct.Commun.* ,**61,**m380

Formula: $C_{16}H_{36}Cl_8Cu_4N_4O_8$

Compound Name: bis(μ_2-Chloro)-hexachloro-tetrakis(μ_2-dimethylglycine-O,O')-tetra-copper(ii)

Space Group: P21/c ***Cell:*** ***a*** 9.759(1) ***b*** 12.861(1) ***c*** 14.060(1)
Space Group No.: 14 ***(Å,°)*** α 90.00 β 99.20(3) γ 90.00

R-Factor (%): 2.35 ***Temperature(K):*** 293 ***Density(g/cm³):*** 1.812

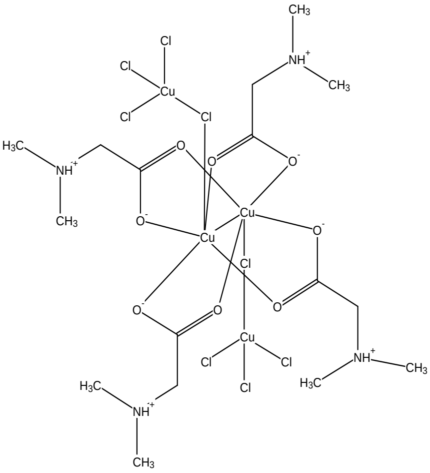

Parameters

Fragment 1
DIST1 (D) 2.730
DIST2 (D) 2.463
DIST3 (D) 1.974
ANG1 (X) 87.527

WEXHOW

Reference: V.Kh.Sabirov, N.M.Kebets, M.A.Porai-Koshits, Yu.T.Struchkov (1994) *Koord.Khim.(Russ.)(Coord.Chem.)* ,**20,**466

Formula: $C_{16}H_{36}Cl_2Cu_2N_4O_8{}^{2+}$,$2(Cl_1{}^{1-})$,$3(H_2O_1)$

Compound Name: tetrakis(μ_2-γ-Ammoniobutyrato-O,O')-dichloro-di-copper(ii) dichloride trihydrate

Space Group: P21 ***Cell:*** ***a*** 11.935(4) ***b*** 9.069(3) ***c*** 14.276(3)
Space Group No.: 4 ***(Å,°)*** α 90.00 β 101.37(2) γ 90.00

R-Factor (%): 4.70 ***Temperature(K):*** 295 ***Density(g/cm³):*** 1.612

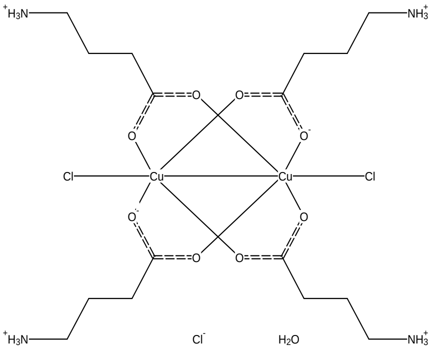

Parameters

Fragment 1
DIST1 (D) 2.649
DIST2 (D) 2.424
DIST3 (D) 1.982
ANG1 (X) 7.102

Search: search5 (Sun Aug 20 15:58:38 2017): Hits 13-16

WUFMIU

Reference: Rui-Bo Xu, Xing-You Xu, Ming-Yan Wang, Xu-Jie Yang, Xin Wang, Lu-De Lu, Wei-Xing Ma (2009) *Jiegou Huaxue(Chin.)(Chin.J.Struct.Chem.)* ,**28,**703

Formula: $C_8 H_{12} Cl_2 Cu_2 O_8{}^{2-}$,2($C_2 H_6 N_1 O_1{}^{1+}$)

Compound Name: 1-Hydroxyethaniminium tetrakis(μ_2-acetato-O,O')-dichloro-di-copper(ii)

Space Group: P21/c ***Cell:*** ***(Å,°)*** ***a*** 8.298(1) ***b*** 14.358(2) ***c*** 12.001(0)
Space Group No.: 14 α 90.00 β 130.62(0) γ 90.00

R-Factor (%): 6.17 ***Temperature(K):*** 298 ***Density(g/cm³):*** 1.696

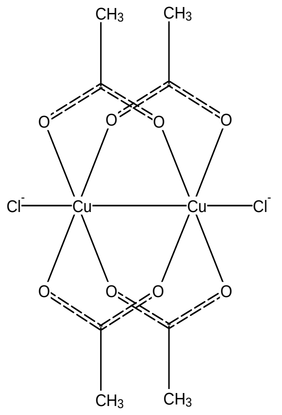

Parameters

Fragment 1
DIST1 (D) 2.654
DIST2 (D) 2.478
DIST3 (D) 1.972
ANG1 (X) 84.456

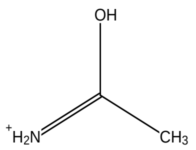

YITGOX

Reference: Xiao-Ming Chen, T.C.W.Mak (1993) *Struct.Chem.* ,**4,**247

Formula: $C_{20} H_{44} Cl_2 Cu_2 N_4 O_8{}^{2+}$,$C_{10} H_{22} Cl_2 Cu_1 N_2 O_4$,2($Cl_1{}^{1-}$)

Compound Name: bis(bis(μ_2-Betainato-O,O')-chloro-copper(ii)) bis(betainato-O)-dichloro-copper(ii) dichloride

Space Group: C2/c ***Cell:*** ***(Å,°)*** ***a*** 30.100 ***b*** 13.533 ***c*** 11.710
Space Group No.: 15 α 90.00 β 103.78 γ 90.00

R-Factor (%): 4.00 ***Temperature(K):*** 295 ***Density(g/cm³):*** 1.586

Parameters

Fragment 1
DIST1 (D) 2.794
DIST2 (D) 2.415
DIST3 (D) 1.963
ANG1 (X) 0.040

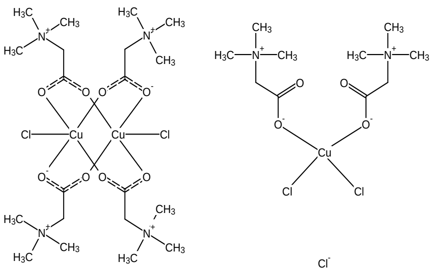

YITGUD

Reference: Xiao-Ming Chen, T.C.W.Mak (1993) *Struct.Chem.* ,**4,**247

Formula: 3($C_{28} H_{28} Cl_2 Cu_2 N_4 O_8{}^{2+}$),2($Cl_4 Cu_1{}^{2-}$),2($Cl_1{}^{1-}$)

Compound Name: tris(tetrakis(μ_2-Pyridylbetainato-O,O')-dichloro-di-copper(ii)) bis(tetrachloro-copper(ii)) dichloride

Space Group: P-3 ***Cell:*** ***(Å,°)*** ***a*** 22.842 ***b*** 22.842 ***c*** 6.999
Space Group No.: 147 α 90.00 β 90.00 γ 120.00

R-Factor (%): 6.80 ***Temperature(K):*** 295 ***Density(g/cm³):*** 1.429

Parameters

Fragment 1
DIST1 (D) 2.795
DIST2 (D) 2.435
DIST3 (D) 1.988
ANG1 (X) 0.020

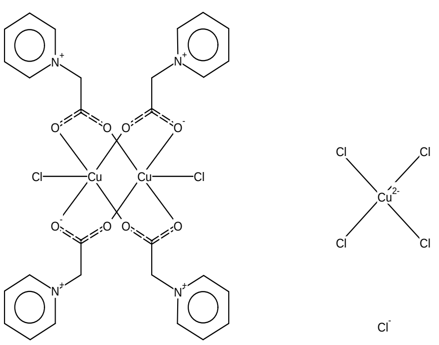

MATDOD

Reference: M.Trzebiatowska-Gusowska, A.Gagor (2017) *J.Coord.Chem.* ,

Formula: $C_4 H_4 Cl_2 Cu_2 O_8{}^{2-}$,$C_4 H_{14} N_2{}^{2+}$

Compound Name: butane-1,4-diaminium dichloro-tetrakis(μ-formato)-di-copper(ii)

Space Group: P21/n ***Cell:*** ***(Å,°)*** ***a*** 7.192(0) ***b*** 11.604(0) ***c*** 9.995(0)
Space Group No.: 14 α 90.00 β 104.20(0) γ 90.00

R-Factor (%): 1.66 ***Temperature(K):*** 295 ***Density(g/cm³):*** 1.923

Parameters

Fragment 1
DIST1 (D) 2.691
DIST2 (D) 2.480
DIST3 (D) 1.975
ANG1 (X) 87.878

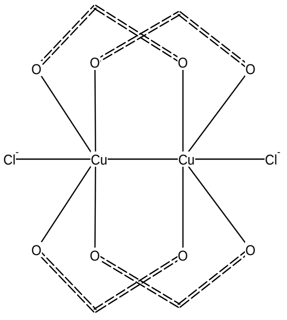

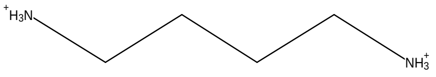

Search: search5 (Sun Aug 20 15:58:38 2017): Hit 17

MATDUJ

Reference:	M.Trzebiatowska-Gusowska, A.Gagor (2017)<br>*J.Coord.Chem.* ,
Formula:	$C_4 H_4 Cl_2 Cu_2 O_8{}^{2-}, C_3 H_{12} N_2{}^{2+}$
Compound Name:	propane-1,3-diaminium dichloro-tetrakis(μ-formato)-di-copper(ii)

Space Group:	P21/n	***Cell:***	***a***	7.031(0)	***b***	11.581(0)	***c***	9.366(0)
Space Group No.:	14	***(Å,°)***	α	90.00	β	99.36(0)	γ	90.00

R-Factor (%): 2.82 ***Temperature(K):*** 295 ***Density(g/cm³):*** 2.005

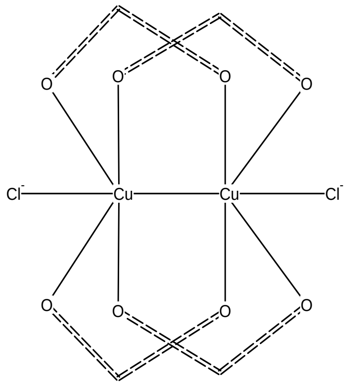

Parameters

Fragment 1

**DIST1 (D)**	2.688
**DIST2 (D)**	2.439
**DIST3 (D)**	1.965
**ANG1 (X)**	87.674

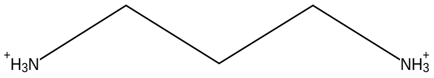

Supplement: Supplementary file 3 [file e-73-01421-sup3.pdf]
